# Protein Adsorption on SiO_2_-CaO Bioactive Glass Nanoparticles with Controllable Ca Content

**DOI:** 10.3390/nano11030561

**Published:** 2021-02-24

**Authors:** Martin Kapp, Chunde Li, Zeqian Xu, Aldo R. Boccaccini, Kai Zheng

**Affiliations:** 1 Institute of Biomaterials, University of Erlangen-Nuremberg, 91058 Erlangen, Germany; martin-kapp@web.de (M.K.); lichunde724@gmail.com (C.L.); aldo.boccaccini@fau.de (A.R.B.); 2 Section Medical Materials Science & Technology, University Hospital Tübingen, 72076 Tübingen, Germany; xuzeqian934130@gmail.com

**Keywords:** bioactive glass nanoparticles, calcium, protein adsorption

## Abstract

Bioactive glass nanoparticles (BGNs) are emerging multifunctional building blocks for various biomedical applications. In this study, the primary aim was to develop monodispersed binary SiO_2_-CaO BGNs with controllable Ca content. We successfully synthesized such spherical BGNs (size ~110 nm) using a modified Stöber method. Our results showed that the incorporated Ca did not significantly affect particle size, specific surface area, and structure of BGNs. Concentrations of CaO in BGN compositions ranging from 0 to 10 mol% could be obtained without the gap between actual and nominal compositions. For this type of BGNs (specific surface area 30 m^2^/g), the maximum concentration of incorporated CaO appeared to be ~12 mol%. The influence of Ca content on protein adsorption was investigated using bovine serum albumin (BSA) and lysozyme as model proteins. The amount of adsorbed proteins increased over time at the early stage of adsorption (<2 h), regardless of glass composition and protein type. Further incubation of BGNs with protein-containing solutions seemed to induce a reduced amount of adsorbed proteins, which was more significant in BGNs with higher Ca content. The results indicate that the Ca content in BGNs is related to their protein adsorption behavior.

## 1. Introduction

Bioactive glass nanoparticles (BGNs) are emerging multifunctional biomaterials in numerous applications, such as bone regeneration, wound healing, and drug delivery [1,2,3]. BGNs usually act as rigid fillers in polymeric matrices (e.g., hydrogels, electrospun polymer fibers) to develop advanced nanocomposites [4,5]. Because of their small size, uniform shape, and abundant surface chemistry for functionalization, BGNs as fillers can lead to more integrated composites with enhanced mechanical performance, bioactivity, and biological activities compared to their microsized counterparts [1,6]. BGNs are also promising delivery platforms of therapeutic agents (e.g., antibiotics, growth factors, biologically active ions) due to their tunable morphology and chemical composition [1,2]. Uniform morphology, desirable particle dispersity, and controllable composition of BGNs are highly related to their performance in the above biomedical applications.

BGNs are usually synthesized using sol–gel processing, a wet chemistry route involving the hydrolysis and condensation of alkoxide precursors (typically tetraethyl orthosilicate) [1]. By varying the sol–gel processing parameters (e.g., the molar ratio of precursors and catalysts), the size, chemical composition, and textural properties of BGNs can be controlled [7,8,9,10,11]. The composition significantly influences the structure and properties of glasses [12]. Particularly, calcium (Ca) is an essential component that distinguishes BGNs from silica nanoparticles, as Ca significantly contributes to the formation of hydroxyapatite (HA) that can bond to bone tissues [7]. The released Ca^2+^ ions can also induce favorable cell activities (e.g., proliferation, osteogenic differentiation, extracellular matrix mineralization) involved in bone regeneration [13,14]. Controlled incorporation of Ca in BGNs is thus important to modulate the physiological and biological activities of BGNs.

One aim of this study was to develop binary SiO_2_-CaO BGNs with controlled Ca content. In our previous work, monodispersed and spherical BGNs with SiO_2_-CaO compositions have been successfully synthesized using a modified Stöber method [10,11]. However, the actual concentration of incorporated CaO was lower than the nominal CaO concentration in these particles. Such a gap between nominal and actual compositions of BGNs has also been reported in the literature [7,8,9]. Given the concentration-dependent therapeutic effects of biologically active ions, precise control over the amount of incorporated CaO is crucial as it is directly related to the ion-released behavior. In the present study, we synthesized BGNs with a particle size of ~110 nm by adjusting the processing parameters in the modified Stöber method developed in our group [10]. The synthesized BGNs remained monodispersed and uniform in size and shape. Importantly, it was revealed for the first time that the gap between actual and nominal compositions of BGNs could be eliminated in a specific concentration range. However, excess CaO beyond this range could not be incorporated into BGNs, thus leading to a composition gap. 

In addition to degradation and mineralization, another important activity also occurs on bioactive glass (BG) surfaces when BGs are in contact with biological fluids containing proteins (e.g., blood plasma and interstitial fluids) or are implanted in vivo [15]. The in vivo host response to an implanted biomaterial is highly related to the adsorbed proteins [16], which plays significant roles in guiding cell behaviors, including adhesion, proliferation, migration, and differentiation [17]. Protein adsorption on nanoparticles can also lead to the formation of protein corona determining their biological fate, which is key for the applications of nanoparticles in nanomedicine [18]. It has been well documented that numerous factors can influence protein adsorption on biomaterials, including environmental characteristics (e.g., temperature, pH), protein properties (e.g., dimension and molecule weight of proteins), and biomaterial characteristics (e.g., hydrophilicity, surface roughness) [17]. Glass composition can also affect protein adsorption on BGs [15]. It is known that the content of CaO in BGNs can affect their degradation and bioactivity [7,8,19]. However, the influence of CaO content in BGNs on protein adsorption has not been investigated yet. Given the emerging applications of BGNs in drug delivery, it is crucial to understand the protein adsorption on this nanoparticle type. In this study, we thus developed a series of BGNs with CaO concentrations ranging from 0 to 10 mol% in the glass composition to investigate the influence of Ca content on protein adsorption using bovine serum albumin (BSA) and lysozyme (LYZ) as model proteins. 

## 2. Materials and Methods

### 2.1. Materials

Tetraethyl orthosilicate (TEOS, ≥99%) was purchased from Sigma-Aldrich (St. Louis, MO, USA). Ethanol (96%), ammonia solution (28%) and calcium nitrate tetrahydrate (CaN) were obtained from VWR International (Radnor, TN, USA). Bovine serum albumin (BSA) (≥96%) and lysozyme from chicken egg white (lyophilized powder, protein ≥ 90%) were purchased from Sigma-Aldrich (St. Louis, MO, USA). Dulbecco’s phosphate-buffered saline (1X) (PBS) and Pierce^TM^ BCA Protein Assay Kit were purchased from Thermo Fisher Scientific Inc. (Waltham, MA, USA). Potassium bromide (KBr) was obtained from Carl Roth (Karlsruhe, Germany). All chemicals were used as received without further purification.

### 2.2. Synthesis of Bioactive Glass Nanoparticles (BGNs)

**Figure 1:**
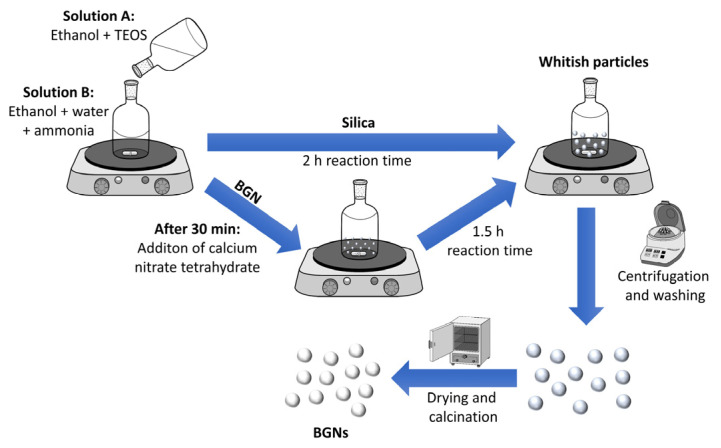
Schematic illustration of the synthesis route for bioactive glass nanoparticles (BGNs).

BGNs were synthesized using a modified Stöber method as reported previously [10,11]. Figure 1 illustrates the schematic of the synthesis procedure. In a typical synthesis process, a solution (A) (TEOS in ethanol) was mixed with a solution (B) that was composed of ammonia, ethanol and deionized water under stirring. After 30 min of reaction, varying amounts (corresponding to the nominal compositions of BGNs) of calcium nitrate tetrahydrate were added. The mixtures were allowed to react for a further 90 min before collection by centrifugation at 7197 rcf (centrifuge 5430 R, Eppendorf AG, Hamburg, Germany) for 25 min. The obtained pellets were dispersed and washed twice with deionized water and once with ethanol. The collected particles were subsequently dried at 60 °C overnight before calcination at 700 °C for 2 h with a heating rate of 2 °C/min. The recipe for synthesizing different BGNs is given in Table 1. BGNs of nominal compositions (100 − *X*) SiO_2_ − *X*CaO (in mol%), *X* = 0, 1, 5, 10, and 30 were synthesized. The obtained nanoparticles were designated as 100S, 99S, 95S, 90S, and 70S, respectively, according to the concentration of CaO in the composition.

### 2.3. Particle Characterization

The morphology of BGNs was characterized by using field-emission scanning electron microscopy (FE-SEM) (Auriga, Zeiss, Oberkochen, Germany) under an accelerating voltage of 2 kV. The particle size was determined by counting particles presented in SEM images using Image J (NIH, Bethesda, MD, USA). The chemical composition of BGNs was determined using Energy-dispersive X-ray spectroscopy (EDS) spectroscopy (X-Max^N^, Oxford Instruments, Abingdon, UK). The zeta potential of BGNs was measured by a Zetasizer Nano ZS (Malvern Instruments, Malvern, UK) instrument with a 4 mW HeNe laser (633 nm) and a light-scattering detector positioned at 90°. The zeta potential values of BGNs were measured in PBS (pH ~7.3, Thermo Fisher Scientific, Waltham, MA, USA) at a concentration of 1 mg/mL. BGNs were also characterized by Fourier transform infrared spectroscopy (FTIR, Nicolet 6700, Thermo Fisher Scientific, Waltham, MA, USA). In the FTIR analysis, BGNs were mixed with KBr at a weight ratio of 1:100 to form pellets for measurements. FTIR spectra were recorded in transmittance mode between 400 and 2000 cm^−1^ with a resolution of 4 cm^−1^. X-ray diffraction (XRD) of BGNs was carried out using a Philips X’pert diffractometer (Philips, Eindhoven, The Netherlands) in the 2*θ* range of 20–80° with Cu Kα radiation. A step size of 0.020° with a dwelling time of 1 s per step was used to record the XRD patterns. The Brunauer–Emmett–Teller (BET) specific surface area of BGNs was measured using the nitrogen sorption method on a Micromeritics porosimeter (ASAP2460, Micrometrics Instrument, Atlanta, GA, USA). Before the BET analysis, BGNs were degassed at 200 °C under vacuum for 6 h. 

### 2.4. Protein Adsorption 

Selected BGNs (100S, 99S, 95S and 90S BGNs) were used for protein adsorption experiments. Bovine serum albumin (BSA) and lysozyme (LYZ) were selected as the model proteins. BSA and LYZ stock solutions were prepared by dissolving proteins in PBS at given concentrations (100, 200 or 500 μg/mL). Afterward, 100 mg of each BGN were dispersed in 10 mL of protein solution in low-protein binding centrifuge tubes (Falcon^®^, St. Louis, MO, USA). All BGN/protein suspensions were then placed in a shaking incubator (KS 4000i control, IKA, Staufen, Germany) at 37 °C and 100 rpm) for predetermined times (0.5, 2, 4, and 24 h). After adsorption for specific times, the samples were immediately collected by centrifugation at 7197× *g* for 10 min. The supernatants of each sample were collected and stored at 4 °C in a fridge for further analysis. After adsorption, the particles were washed once with PBS to remove loosely bound protein molecules and dried at 60 °C overnight for further analysis. Protein solutions without particles were prepared as references. All experiments were carried out at least in triplicate.

To determine the amount of adsorbed proteins, we analyzed the concentrations of proteins (BSA or LYZ) in the supernatants after the adsorption by using the bicinchoninic acid (BCA) assay (Pierce^TM^ BCA Protein Assay Kit, Thermo Fisher Scientific, Waltham, MA, USA) following the manufacturer’s instructions. In brief, 50 μL of supernatants from each group were mixed with 1 mL of BCA working solution and stored in the shaking incubator for 30 min at 37 °C until a purple color was observed. References without proteins (only PBS) were prepared as a control. The protein concentration of supernatants was quantified by a UV-vis spectrophotometer (Specord 40, Analytik Jena, Jena, Germany) at 562 nm. The values of adsorbed protein concentrations were calculated by subtracting the measured protein concentration of supernatants from the initial concentration of protein solution. All samples were prepared in triplicate and measured three times on the same day. Moreover, the values of the measured protein concentrations were normalized to the BSA or LYZ concentrations used for the references. 

### 2.5. Statistical Analysis

The results were shown as mean ± standard deviation (SD). Statistical evaluation was assessed by one-way analysis of variance (ANOVA) and post hoc Tukey’s test. *p* < 0.05 was considered statistically significant.

## 3. Results and Discussion

### 3.1. Synthesis and Morphology of BGNs

**Figure 2:**
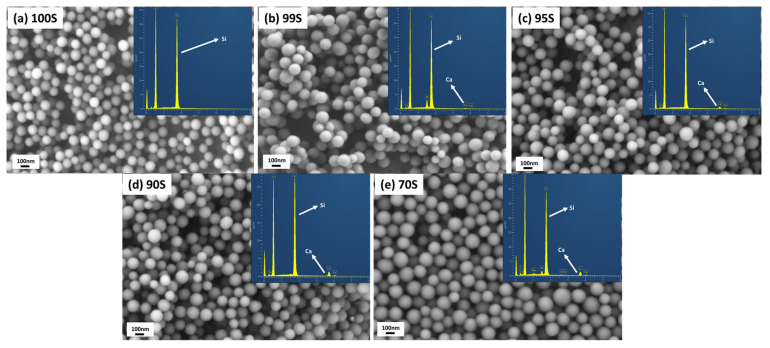
Scanning electron microscopy (SEM) images of (**a**) 100S, (**b**) 99S, (**c**) 95S, (**d**) 90S and (**e**) 70S (inserted corresponding Energy-dispersive X-ray spectroscopy (EDS) spectra).

In this study, BGNs were synthesized using a modified Stöber method, a well-established approach towards monodispersed silicate nanoparticles [10,20]. We have developed relatively large SiO_2_-CaO BGNs (~400 nm) using this approach as reported in our previous studies [10,21]. Figure 2 shows SEM images of the synthesized BGNs with different nominal compositions. All nanoparticles showed a spherical shape with a smooth and nonporous surface, characteristic morphology of nanoparticles synthesized by the Stöber process [9,11]. All nanoparticles exhibited similar particle sizes of ~110 nm regardless of the amount of Ca precursor (CaN) added. It is known that the particle size of BGNs can be tailored by tuning processing parameters in the Stöber process, such as the molar ratio of ethanol/water and catalyst concentration [9,22,23]. We adjusted the molar ratio of water/ethanol and the concentration of TEOS used in comparison to the parameters applied in our previous study [10], leading to smaller particles (~110 nm). Kesse et al. [9] have reported that the variation of the initial Ca/Si molar ratio had no significant impact on the size of the resulting particles. Our results agreed with their finding that the increased molar ratio of CaN/TEOS did not significantly change the particle size of the resulting BGNs. However, it is known that the timing of the addition of CaN can indeed influence colloidal stability, particle size and dispersity of resulting BGNs [9,10]. In the present study, the timing of CaN addition was decided based on the optimization in our previous study [10], which led to monodispersed BGNs with uniform particle size and shape (Figure 2).

The specific surface area (SSA) of 100S, 99S, 95S, 90S, and 70S were measured to be 34.4, 30.2, 31.3, 29.7, and 27.2 m^2^/g, respectively (Table 2), which were in agreement with the SSA values of nonporous particles with a size of ~100 nm reported in the literature [9,23]. It is known that silicate nanoparticles synthesized by the Stöber method possess microporosity before heating treatment [24]. However, this microporosity can be eliminated by high-temperature treatment (e.g., 700 °C) [25]. In our synthesis, 700 °C was selected as the calcination temperature to ensure the diffusion of Ca into the silicate network as well as the thermal decomposition of nitrates from the Ca precursor [26]. The calcinated BGNs thus exhibited dense and nonporous morphology, also evidenced by their relatively low SSA (Table 2). Given the similar particle size and nonporous structure, it is understandable that no significant difference in SSA could be observed among these BGNs. Also, all nanoparticles exhibited negative zeta potential values (~−24 mV) in PBS (Table 2), indicating their negatively charged surface in physiological fluids. It can thus be concluded that the amount of added CaN (Ca precursor) did not significantly affect the morphology of resulting BGNs in this modified Stöber process when the timing of CaN addition was certain.

### 3.2. Incorporation of Calcium into BGNs

The chemical composition of sol–gel-derived BGNs can be tuned by adjusting the type and amount of added precursors in sol–gel processing [9,10,23]. We modified the composition of BGNs by varying the amount of added CaN. Figure 2 shows EDS spectra of the synthesized BGNs. Their calculated compositions are given in Table 2. As expected, peaks related to Ca were observed in EDS spectra of BGNs except for 100S. A higher intensity of Ca peak could be observed in the spectra of BGNs with a larger amount of CaN added. The calculated concentrations of CaO in 100S, 99S, 95S, and 90S were 0, 1, 4.9, and 10.4 mol%, respectively, close to their nominal compositions. However, the calculated concentration of CaO in 70S was 12.3 mol%, far lower than the nominal one (30 mol%). 

**Figure 3:**
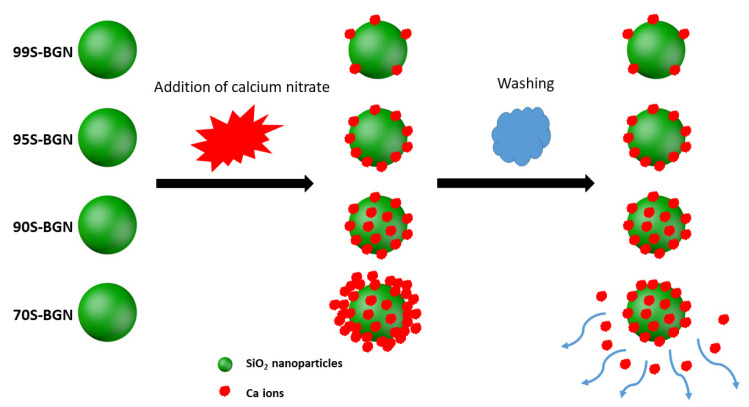
Schematic illustration of the mechanism of Ca incorporation into BGNs in the Stöber method.

It has been well documented that a gap exists between the nominal and actual compositions of BGNs synthesized using the Stöber-based method [9,10]. In the Stöber process, Ca^2+^ ions are first adsorbed onto the surface of silica nanoparticles and enter the silicate network during the heating treatment [10,26]. The capacity of particle-adsorbing Ca^2+^ ions is the main factor determining the Ca content in the resulting BGNs, which mainly depends on the surface chemistry and SSA of silica nanoparticles [9,11,23]. It seems that in the synthesis of 99S, 95S, and 90S, the primary silica nanoparticles could adsorb almost all added Ca^2+^ ions, suggesting that the amount of added Ca^2+^ ions did not exceed their adsorbing capacity. As a comparison, although 70S exhibited a slightly higher concentration of CaO than 90S (Table 2), its actual CaO concentration (~12 mol%) was still far lower than the designed one (30 mol%). It could be deduced that this concentration (~12 mol%) of CaO could have reached the maximum amount of Ca ions that could be adsorbed by this type of nanoparticles (~110 nm of particle size, ~30 m^2^/g of SSA). Therefore, in the case of 70S synthesis, excess Ca^2+^ ions were not incorporated into the nanoparticles, leading to a composition gap in the resulting BGNs. Notably, it seems that the adsorbed Ca^2+^ ions were not significantly removed from the particles during washing steps in the synthesis, as indicated by the comparable concentrations of CaO between the actual and nominal compositions in 99S, 95S, and 90S. However, the washing step could still influence the final content of CaO in BGNs by removing excess adsorbed Ca^2+^ ions (e.g., in the case of 70S) [10]. Figure 3 shows the illustration of the synthesis process and a possible explanation to the composition differences in various BGNs. In the cases of 99S, 95S, and 90S, the silica nanoparticles possessed sufficient active sites to adsorb Ca^2+^ ions. The following washing step was not able to remove the adsorbed ions. Adversely, in the case of 70S, excess Ca^2+^ ions could not be adsorbed on the surface of the nanoparticles. Some Ca^2+^ ions might be loosely bonded with the outer layer of the particles, which could be easily removed by the washing step. 

Taken together, we can conclude that the content of incorporated CaO in binary SiO_2_-CaO BGNs depends on the surface chemistry and SSA of primary SiO_2_ nanoparticles. A maximum content of CaO that can be incorporated into BGNs is available for specific BGNs (with certain topography and surface chemistry). In this study, the actual concentrations of CaO incorporated in 99S, 95S, and 99S were close to their nominal concentrations. 70S possessed the maximum CaO concentration (~12 mol%) for this type of nanoparticles, which was still far lower than the nominal concentration of CaO (30 mol%). The difference in the amount of added CaN (Ca precursor) did not significantly affect the particle size, shape, and colloidal stability of BGNs. Given the comparative morphology and surface charge of these BGNs, their protein adsorption behavior was expected to be mainly influenced by the difference in CaO content. 100S, 99S, 95S, and 90S were thus selected for further characterization and protein adsorption experiments.

### 3.3. Structural Analysis of BGNs

**Figure 4:**
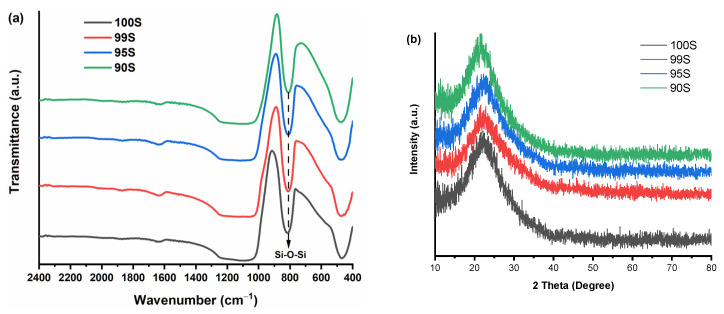
(**a**) Fourier transform infrared spectroscopy (FTIR) spectra and (**b**) X-ray diffraction (XRD) patterns of 100S, 99S, 95S, and 90S.

Figure 4a shows FTIR spectra of 100S, 99S, 95S, and 90S. No significant difference could be observed among the spectra of different BGNs. All nanoparticles exhibited typical FTIR bands of silicate-based glasses. The bands located at 468 and 810 cm^−1^ were related to the Si–O–Si rocking mode and the Si–O–Si symmetric stretching mode, respectively [10,27]. The broad band located between 1035 and 1240 cm^−1^ could be assigned to the Si–O–Si asymmetric stretching mode [27]. Furthermore, a small band located at approximately 1636 cm^−1^ was associated with the vibration of hydroxyl groups, probably due to the presence of water on BGNs [10]. In previous studies, solid-state nuclear magnetic resonance (solid NMR) analysis has evidenced that Ca can enter the silicate network of SiO_2_-CaO BGNs produced by the Stöber method and act as a network modifier [10,23]. In 99S, 95S, and 90S, Ca could also enter the silicate structure of BGNs. However, due to the relatively low amounts of Ca incorporated, no significant changes could be detected in the FTIR spectra of nanoparticles before and after Ca incorporation [10]. XRD analysis (Figure 4b) confirms the amorphous characteristic of all BGNs after calcination at 700 °C as no diffraction peaks were observed in XRD patterns. The above results indicated that the structure of the particles was not significantly affected by the incorporation of Ca regardless of its amount.

### 3.4. Effects of CaO Content in BGNs on Protein Adsorption

Given the similar particle size, surface charge, and SSA, the difference in protein adsorption on 100S, 99S, 95S, and 90S was assumed to be induced by their different CaO contents. BSA was selected as a model protein for this study given the abundant presence of albumin in serum proteins [28]. We first investigated the influence of BSA concentration on protein adsorption on BGNs. Three concentrations of BSA, i.e., 100, 200, and 500 µg/mL, were used for the test. The concentration of adsorbed BSA on BGNs was recorded after 2 h of soaking in BSA solutions, as this time period was expected to have a considerable amount of adsorbed protein for detection according to our previous study [19]. 

**Figure 5:**
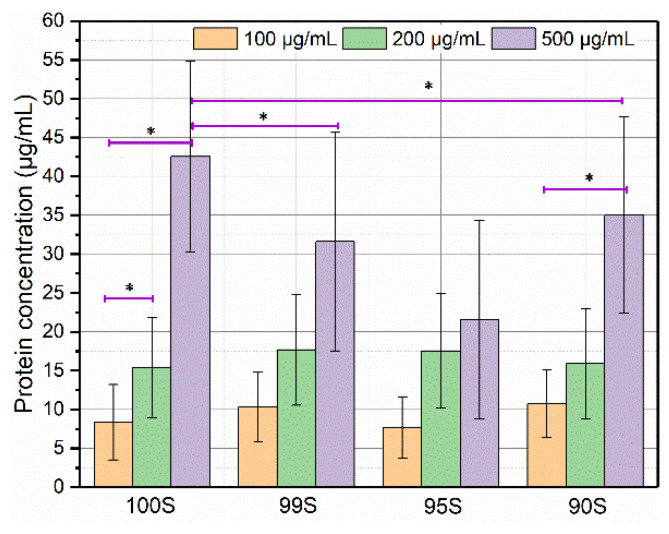
The concentrations of bovine serum albumin (BSA) adsorbed on 100S, 99S, 95S, and 90S after incubation in BSA solutions for 2 h at different BSA concentrations (100, 200, and 500 μg/mL, respectively). Three technical repeats (*n* = 3) were carried out within each experiment that was repeated in triplicate (*N* = 3). Data are presented as mean ± SD and * indicates *p* < 0.05.

Figure 5 shows the concentrations of adsorbed BSA on 100S, 99S, 95S, and 90S after incubation in BSA solutions of different BSA concentrations. As expected, when a higher concentration of BSA solution was used, a larger amount of BSA could be adsorbed on BGNs regardless of glass compositions (typically in 100S and 90S). This phenomenon was in agreement with the results reported in a previous study [29]. At relatively low concentrations (100 and 200 µg/mL), no significant difference in BSA adsorption could be observed among different nanoparticles. However, at the concertation of 500 µg/mL, 100S adsorbed the largest BSA amount compared to Ca-containing BGNs. It seems that the presence of Ca in the particles reduced the amount of adsorbed BSA. However, we should consider that the specific surface area (another important factor influencing protein adsorption) of the particles is (slightly) different. We thus calculated the amount of adsorbed BSA for unit surface area in BGNs. 100S, 99S, 95S, and 90S adsorbed 1.26, 1.07, 0.71, and 1.17 μg·mL^−1^/m^2^·g^−1^, respectively, which indicated that the incorporation of Ca in the particles also reduced the amount of adsorbed BSA per unit surface area. We thus hypothesized that the presence of Ca in BGNs could be the main factor affecting protein adsorption and selected the concentration of 500 µg/mL for the protein adsorption experiments on BGNs over time.

In this study, an indirect method by measuring the protein concentration of supernatant after incubation with BGNs was applied to determine the amount of adsorbed proteins. This method has been widely used to evaluate the protein adsorption on bioceramics and BGs [30,31]. However, it should be pointed out that this method has some limitations. One of the limitations is that protein could also likely adsorb to the container the experiment is conducted within. Thus, longer incubation could deplete more protein from the solution regardless of the presence of materials. To minimize this influence, we selected low-protein binding centrifuge tubes for incubating BGNs in protein solutions. Moreover, proteins could also precipitate over time and fall out of solution. It is known that protein precipitation could be affected by ionic strength and pH of solution that are dynamically changing over time in a solution containing BGNs [32]. Due to the high surface reactivity, degradation and surface mineralization of BGNs could take place rapidly after their immersion in physiological fluids, which induce the change in ionic concentration and pH values of solution [1]. The content of Ca in BGNs can affect their degradation and mineralization, consequently affecting protein precipitation. However, we could consider that the potential protein precipitation over time in a relatively long incubation time (24 h) was also influenced by the Ca content in BGNs. 

**Figure 6:**
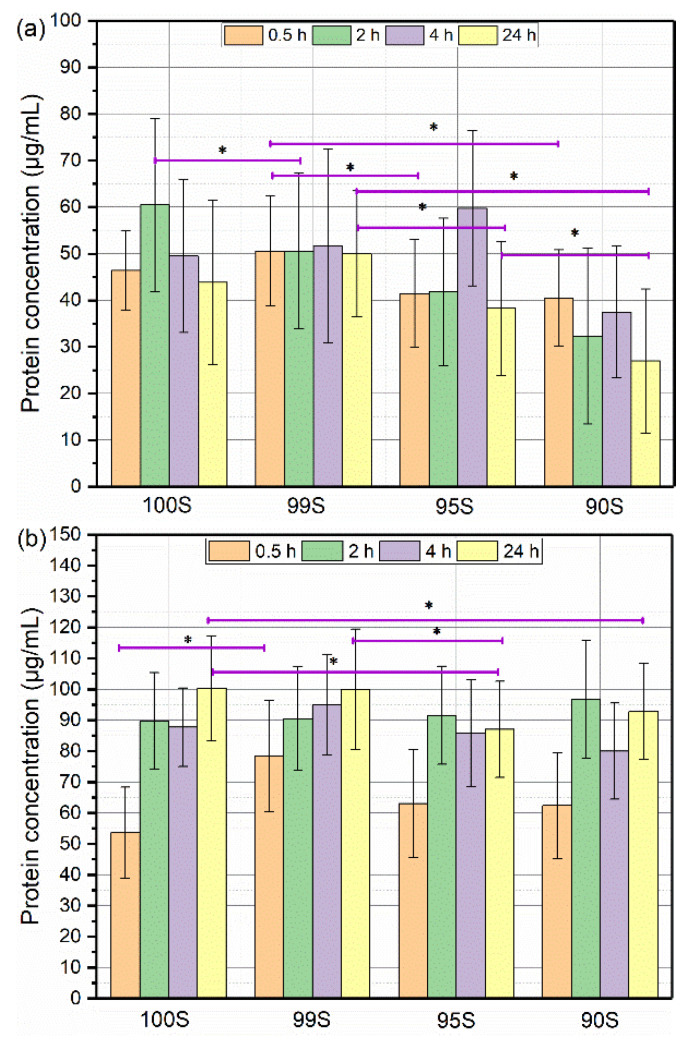
The concentrations of (**a**) BSA (**b**) lysozyme (LYZ) adsorbed on various BGNs after incubation for 0.5 h, 2 h, 4 h, and 24 h. Three technical repeats (*n* = 3) were carried out within each experiment that was repeated in triplicate (*N* = 3). Data are presented as mean ± SD and * indicates *p* < 0.05.

To investigate the influence of CaO in BGNs on protein adsorption over time, we selected two model proteins with the opposite surface charge at the physiological pH for the experiment, i.e., BSA and LYZ that have acidic and alkaline isoelectric points (IEPs), respectively. The IEP of BSA is located in a range from 4.7 to 5.3, whereas that of LYZ is located between 10.7 and 11.3 [30]. Therefore, BSA exhibits a negative charge, while LYZ shows a positive charge at the physiological pH 7.4, but both proteins have positive- and negative-charged domains in their molecules [17]. Figure 6 shows the results of protein adsorption on BGNs over time. After 30 min incubation, no significant difference could be observed between 100S and 99S (Figure 6a), whereas 95S and 90S seemed to adsorb less BSA than 100S and 99S (but no significant difference between 95S and 90S). After incubation in the BSA solution for 2 h, the amount of adsorbed BSA on 100S increased, while those on 99S, 95S, and 90S were not significantly changed. However, 100S adsorbed a considerably larger amount of BSA than 9SS, 95S, and 90S. Also, the amount of adsorbed BSA on 99S was significantly higher than those on 90S and 95S. After incubation for 4 h, a significant increase in the amount of adsorbed BSA was observed on 95S. The amount of adsorbed BSA remained stable for most BGNs after 24 h of adsorption, though a slight reduction in the amount of adsorbed BSA was observed on 90S. Compared to 100S and 99S, 90S and 95S that possessed relatively larger contents of CaO seemed to adsorb a lower amount of BSA after 24 h incubation. Similar to the findings in BSA adsorption, LYZ adsorption results (Figure 6b) showed that the amount of LYZ on BGNs increased in the first 2 h of incubation and then reached almost an equilibrium, which agreed with the results reported in a previous study [33]. After 24 h incubation, it seemed that the amounts of adsorbed LYZ on 95S and 90S were lower than those on 100S and 99S. It appears that a larger amount of LYZ could be adsorbed on BGNs in comparison to the adsorption of BSA (Figure 6). For example, approximately 60 mg/mL of BSA were adsorbed on 100S, while 100 mg/mL of LYZ could be adsorbed on 100S. This difference could be explained by the lower molecule weight of LYZ and the opposite surface between LYZ and BGNs [30]. LYZ is positively charged at physiological pH while BSA is negatively charged. The electrostatic interactions between LYZ and the negatively charged BGNs were therefore stronger, resulting in more significant adsorption of LYZ molecules [34]. However, besides electrostatics, many other forces can also drive protein adsorption, such as hydrophobic forces. Also, protein molecules have both positively and negatively charged domains, which means that nanoparticles can also adsorb protein molecules even if they have the same net surface charges. 

A variety of factors can affect protein adsorption on BGs [15]. In addition to external factors (e.g., temperature, pH), the internal characteristics of BGs (e.g., nanotopography, surface chemistry) also play important roles in determining the amount and structure of adsorbed proteins [15,17]. Among various intrinsic characteristics, the chemical composition of BGNs affects their properties, such as surface chemistry, surface charge, and surface topography [15]. Notably, the composition also greatly influences the degradation and surface mineralization (apatite formation) of BGNs. Interactions between BGs and proteins have been investigated to understand the protein adsorption kinetic, factors affecting protein adsorption, and the influence of adsorbed proteins on BG properties [15]. For example, we recently reported that protein adsorption on 45S5 BGs is affected by the medium pH [28]. Also, adsorbed serum proteins seem to be able to delay HA formation on BGs [35,36]. Moreover, the influence of bulky BG surface characteristics (e.g., zeta potential, crystallization, porosity) on protein adsorption has been focused on [37,38,39,40]. However, interactions between BGNs and proteins have not been widely investigated before. It is known that protein adsorption on nanoparticles occurs rapidly when the particles are in contact with protein-containing solutions [17,41]. In this study, it seems that the amount of adsorbed proteins on the nanoparticles reached equilibrium after 2 h incubation in the protein solution. Such protein adsorption saturation on silicate nanoparticles has also been observed in a previous study [42]. In relatively sTable 100S and 99S (due to low concentrations of CaO), the amount of adsorbed proteins remained stable within 24 h in protein solutions. However, the amount of adsorbed proteins on 95S and 90S seemed to fluctuate. As elaborated above, in addition to protein adsorption, potential protein precipitation could also contribute to the change of protein concentrations. However, it is challenging to separate the protein adsorption and precipitation in the current study. 

Nevertheless, 95S and 90S, particularly 90S, adsorbed a lower amount of BSA than 100S and 99S at specific incubation time points (Figure 6a). Given the comparable SSA, surface charge, topography, and surface chemistry of all BGNs, this difference in protein adsorption was probably induced by their composition difference (Ca content). In binary SiO_2_-CaO BGNs, the bioactivity (HA formation) increases with the concentration of Ca incorporated [7]. Due to the relatively higher content of Ca in 90S, ion exchange, degradation and formation of an amorphous Ca-P phase could take place on 90S faster than that on 99S and 100S during their soaking in physiological fluids. These activities lead to a significant change in surface charge, composition, hydrophilicity, crystallinity, and topography on BGN surfaces [28,43,44]. All these changes together lead to the relatively low amount of BSA adsorption on 90S compared to other BGNs. However, the exact mechanisms behind this phenomenon are still not clear. More experiments are still required to elucidate the mechanism behind the influence of Ca in BGNs on protein adsorption behavior, not only on the amount of proteins but also on the structure of adsorbed proteins. 

## 4. Conclusions

In this study, we successfully synthesized binary SiO_2_-CaO BGNs using a modified Stöber method. The synthesized BGNs exhibited a spherical shape with a uniform size of ~110 nm. The difference in the amount of added Ca precursor (CaN) did not affect the morphology of the resulting BGNs. The Ca content in BGNs could be controlled by tuning the amount of CaN added. A gap between the actual and nominal compositions was not observed when the incorporated Ca concentrations ranged from 0 to 10 mol% in glass composition. For this type of BGNs (size ~110 nm, specific surface area ~30 m^2^/g), the maximum content of Ca that could be incorporated was ~12 mol%. A gap between nominal and actual composition could be observed when the nominal Ca concentration exceeds 12 mol% for this type of BGNs. The presence of Ca seemed to affect protein adsorption on BGNs at the physiological pH. BGNs (95S, 90S) with relatively higher Ca contents adsorbed a lower amount of BSA than those with lower Ca contents (99S and 100S). This work indicates that the amount of added Ca precursors is the primary reason leading to the gap between nominal and actual compositions of BGNs. In a specific concentration range, SiO_2_-CaO BGNs without a composition gap can be obtained. The results also contribute to understanding the complex and dynamic interactions between proteins and BGNs as well as the influence of Ca content on protein adsorption behavior.

## Figures and Tables

**Table 1:** Nominal composition of BGNs with various CaO concentrations and their corresponding synthesis recipe. TEOS: tetraethyl orthosilicate; CaN: calcium nitrate tetrahydrate.

Nominal Composition (mol%)	Solution A		Solution B			
	TEOS (mL)	Ethanol (mL)	Ammonia (mL)	Ethanol (mL)	Water (mL)	CaN (g)
100SiO_2_ (100S)	12	48	18	33	100	-
99SiO_2_-1CaO (99S)	12	48	18	33	100	0.12
95SiO_2_-5CaO (95S)	12	48	18	33	100	0.66
90SiO_2_-10CaO (90S)	12	48	18	33	100	1.45
70SiO_2_-30CaO (70S)	12	48	18	33	100	5.80

**Table 2:** Nominal and calculated compositions of BGNs determined by EDS results as well as the zeta potential and specific surface area.

Sample Codes	Nominal Composition (mol%)		Calculated Composition (mol%)		Zeta-Potential	Specific Surface Area
	SiO_2_	CaO	SiO_2_	CaO	(mV)	(m^2^/g)
100S	100	0	100	0	−24 ± 1	34.4
99S	99	1	99.0	1.0 ± 0.2	−24 ± 1	30.2
95S	95	5	95.1	4.9 ± 0.2	−25 ± 1	31.3
90S	90	10	89.6	10.4 ± 0.6	−25 ± 1	29.7
70S	70	30	87.7	12.3 ± 1.2	−24 ± 1	27.2

## Data Availability

The data used to support the findings of this study are available from the corresponding author on request.
